# Short-Term Use of Parenteral Nutrition With a Lipid Emulsion Containing a Mixture of Soybean Oil, Olive Oil, Medium-Chain Triglycerides, and Fish Oil: A Randomized Double-Blind Study in Preterm Infants

**DOI:** 10.1177/0148607111424411

**Published:** 2012-01

**Authors:** Maissa Rayyan, Hugo Devlieger, Frank Jochum, Karel Allegaert

**Affiliations:** 1 Department of Neonatology, University Hospitals, Leuven, Belgium; 2 Zentrum für Neonatologie und Pädiatrische Intensivmedizin, Greifswald, Germany. Dr Jochum’s current affiliation is the Department of Pediatrics, Ev. Waldkrankenhaus Spandau, Berlin, Germany

**Keywords:** parenteral nutrition, premature infant, fish oils, triglycerides, liver function, fatty acids

## Abstract

*Background*: For premature neonates needing parenteral nutrition (PN), a balanced lipid supply is crucial. The authors hypothesized that a lipid emulsion containing medium-chain triglycerides (MCTs) and soybean, olive, and fish oils would be as safe and well tolerated as a soybean emulsion while beneficially influencing the fatty acid profile. *Methods*: Double-blind, controlled study in 53 neonates (<34 weeks’ gestation) randomized to receive at least 7 days of PN containing either an emulsion of MCTs and soybean, olive, and fish oils or a soybean oil emulsion. Target lipid dosage was 1.0 g fat/kg body weight [BW]/d on days 1–3, 2 g/kg BW/d on day 4, 3 g/kg BW/d on day 5, and 3.5 g/kg BW/d on days 6–14. *Results*: Test emulsion vs control, mean ± SD: baseline triglyceride concentrations were 0.52 ± 0.16 vs 0.54 ± 0.19 mmol/L and increased similarly in both groups to 0.69 ± 0.38 vs 0.67 ± 0.36 on day 8 of treatment (*P* = .781 for change). A significantly higher decrease in total and direct bilirubin vs baseline was seen in the test group compared with the control group *P* < .05 between groups). In plasma and red blood cell phospholipids, eicosapentaenoic acid and docosahexaenoic acid were higher, and the n-6/n-3 fatty acid ratio was lower in the test group (*P* < .05 vs control). *Conclusions*: The lipid emulsion, based on a mixture of MCTs and soybean, olive, and fish oils, was safe and well tolerated by preterm infants while beneficially modulating the fatty acid profile.

## Clinical Relevancy Statement

Intravenous lipid emulsions are an integral part of the parenteral nutrition regimen in preterm neonates. A substantial body of evidence indicates that a well-balanced fatty acid supply during the neonatal period is a crucial factor influencing outcome criteria such as growth, visual development, and cognitive development. In this study, a lipid emulsion containing fish oil, olive oil, medium-chain triglycerides, and soybean oil was found to be well tolerated in preterm infants, and n-3 long-chain polyunsaturated fatty acids (LC-PUFAs) were significantly increased. The higher availability of n-3 LC-PUFAs with this lipid might be considered advantageous with regard to their postulated role in ensuring adequate brain and retinal development in preterm infants and the postulated anti-inflammatory and immunomodulatory role.

## Introduction

Preterm neonates, having missed the crucial period of intrauterine nutrient accretion and storage, possess only limited energy and fat reserves.^1,2^ When enteral feeding is not tolerated or insufficient to meet the requirements, parenteral nutrition (PN) must be instituted shortly after birth.^2^ Intravenous lipid emulsions are an integral part of the PN regimen in neonates.^2-4^ A substantial body of evidence indicates that a well-balanced fatty acid supply during the neonatal period is a crucial factor influencing outcome criteria such as growth, visual development, and cognitive development.^2,5,6^ Yet, most commonly used soybean oil–based lipid emulsions contain high amounts of linoleic acid (LA; C18:2 n-6) relative to α-linolenic acid (α-LNA; C18:3 n-3) but low amounts of arachidonic acid (AA; C20:4 n-6) and no n-3 long-chain polyunsaturated fatty acids (LC-PUFAs) such as eicosapentaenoic acid (EPA; C20:5 n-3) and docosahexaenoic acid (DHA C22:6 n-3). The need for more appropriate lipid emulsions in pediatric and neonatal care, however, has been recognized.^7^ In enteral feeding studies, LC-PUFAs have been shown to positively influence neurological and mental development in both preterm and term infants.^8-10^ It has been demonstrated that feeding preterm infants formulas containing DHA and AA enhances growth and improves mental and psychomotor development scores as well as visual acuity.^8,11^ EPA functions as a precursor for the LC–fatty acid synthesized in the retina.^12^ Consequently, it has been postulated that LC-PUFAs are important for growth and development of preterm and term infants.^5^

In the present prospective, double-blind, randomized, parallel, controlled study, the safety, tolerability, and efficacy of a test lipid emulsion (SMOFlipid 20%; Fresenius Kabi, Bad Homburg, Germany) were evaluated in premature neonates as compared to a conventional soybean oil emulsion. The emulsion under investigation contains a physical mixture of 4 different lipid sources: soybean oil providing LA and α-LNA for essential fatty acid supply; olive oil rich in monounsaturated fatty acids (MUFAs), which are less susceptible to lipid peroxidation than PUFAs; medium-chain triglycerides (MCTs) showing a faster metabolic clearance than long-chain triglycerides; and fish oil for the supply of n-3 LC-PUFA EPA and DHA.^13-17^

Our primary hypothesis was that the test treatment would be as safe and well tolerated as a standard soybean emulsion with regard to the serum concentration of triglycerides, hematological and clinical laboratory parameters, adverse events (AE) profile, tolerability, and vital signs. Furthermore, we intended to provide documentary evidence that the test emulsion is equally efficient as a standard soybean emulsion in promoting the neonates’ growth while beneficially influencing the fatty acid profile in plasma and red blood cell (RBC) phospholipids.

## Methods

### Patients and Randomization

Between November 2004 and February 2006, 53 premature neonates were enrolled at the Department of Neonatology of the University Hospital Leuven, Belgium. Study protocol, patient information, and informed consent forms were approved by the local Ethical Committee of the University Hospitals, Leuven, Belgium. The study was performed in accordance with the International Conference on Harmonisation (ICH) guidelines for Good Clinical Practice (GCP), the legal provisions of the current version of the local drug law, the European Community GCP-Guidelines, and the ethical principles of the current version of the Declaration of Helsinki as revised in Edinburgh 2000.

Infants were randomly assigned to one of the treatment groups following the pattern of a randomization list generated prior to study start by using the software RANCODE by idv (Institute for Data Analysis and Trial Planning, Gauting, Germany). Within each block, the number of infants allocated to each of the 2 treatments was balanced. The randomization was stratified with regard to birth weight at the time of inclusion (500–1000 g, 1001–1500 g, 1501–2000 g). Infants with a gestational age <34 weeks, birth weights between 500 and 2000 g, and an estimated requirement of PN of at least 7 days were to be included. Adhering to the general safety considerations, we excluded extremely immature neonates and/or those with the following abnormalities requiring therapeutic interventions that would interfere with the treatment regimen: severe congenital malformations, congenital heart failure, anuria due to circulation failure, liver/hemolytic disease, thrombocytopenia, oxygen saturation SO_2_ <80% longer than 2 hours, previous inclusion in other trials, severe acidosis, application of catecholamines, multiorgan failure, or hypoxic-ischemic encephalopathy.

Written informed consent was obtained from the parents before enrollment; parents received detailed information about aims and risks of the study and had the right to withdraw their child from the study at any time.

### Interventions

Infants received PN containing either SMOFlipid 20% (test group; n = 26) or a conventional soybean oil emulsion (Intralipid 20%, control group; n = 27), both from Fresenius Kabi, for at least 7 consecutive days and up to 14 days. To ensure blinding, the study and control infusions were of the same size and identical appearance. Infusions prepared in the hospital pharmacy and provided to the unit could be identified only by the patient number printed on the outside of the packaging. The PN regimens were infused via peripheral vein (n = 4), central venous catheter (CVC; n = 43), or peripheral vein and CVC (n = 6). Eligible patients should have received the 20% lipid emulsions separately as 1 component of PN by continuous infusion at a maximum rate of 0.17 g fat/kg body weight [BW]/h over a recommended period of 20–24 hours per day.^2,4^ Yet, according to patients’ individual requirements and because of hospital routines, the actual period of infusion amounted to 18 h/d. This regimen started on study day 1 and was continued for at least 7 consecutive days and up to 14 days.

The daily target dosage of fat started at 1.0 g fat/kg BW/d on days 1–3 and was increased to 2 g/kg BW/d on day 4, 3 g/kg BW/d on day 5, and 3.5 g/kg BW/d from day 6 on. Table 1 shows the parenteral fat intake day by day. The maximum dose of 3.5 g/kg BW/d is in accordance with the recommendations for maximum fat intake in infants as published by the European Society for Clinical Nutrition and Metabolism (ESPEN)/European Society of Paediatric Gastroenterology, Hepatology, and Nutrition (ESPGHAN) in 2005.^2^ When serum triglyceride levels exceeded 300 mg/dL (3.43 mmol/L), the dosage was reduced by steps of 0.5 g fat/kg BW according to the clinical condition. Composition and α-tocopherol content of the investigational products are given in Table 2. Fatty acid profiles of the lipid compositions under investigation are provided in Table 3. Other components of PN were given as standardized solutions at the discretion of the investigator.^18^ Additional enteral intake comprising <30% of the total lipid intake on days 1–3, <50% on days 4–7, and <70% on days 8–14 of the total energy intake was permitted if appropriate and documented.

**Table 1.** Parenteral Fat Intake (g/kg Body Weight), Intent-to-Treat Population

	n	Test Group (SMOFlipid)	n	Control Group
Day 1	26	1.0 (0.2)	27	1.0 (0.1)
Day 2	26	1.0 (0.1)	27	1.0 (0.2)
Day 3	25	1.0 (0.2)	26	1.0 (0.2)
Day 4	24	2.0 (0.2)	25	2.0 (0.2)
Day 5	24	2.9 (0.4)	25	3.0 (0.4)
Day 6	24	3.4 (0.5)	23	3.3 (0.5)
Day 7	23	3.3 (0.5)	23	3.3 (0.8)
Day 8	8	3.3 (0.2)	11	3.3 (0.2)
Day 9	8	3.3 (0.3)	9	3.3 (0.2)
Day 10	6	2.9 (1.1)	8	3.3 (0.2)
Day 11	5	3.2 (0.1)	6	3.2 (0.2)
Day 12	5	3.1 (0.1)	6	3.2 (0.3)
Day13	3	3.0 (0.2)	4	3.2 (0.3)
Day 14	3	3.1 (0.3)	2	3.2 (0.6)

Values are presented as mean (SD).

**Table 2.** Composition of SMOFlipid 20% and Intralipid 20%

	SMOFlipid 20%	Intralipid 20%
Soybean oil, g/L	60	200
Medium-chain triglycerides, g/L	60	—
Olive oil, g/L	50	—
Fish oil, g/L	30	—
Vitamin E, mg α-tocopherol/L	Approx. 200	38
Egg phospholipids, g/L	12	12
Glycerol, g/L	25	22.5
Water for injection	ad 1000 mL	ad 1000 mL
PH value	7.5–8.8	7–8
Osmolarity, mosmol/L	273	265

Reprinted with permission from Fresnius Kabi.

**Table 3.** Fatty Acid Profiles of SMOFlipid 20% and Intralipid 20%^a^

	SMOFlipid 20%	Intralipid 20%
Caprylic acid C 8:0	16.3	—
Caproic acid C 10:0	11.4	—
Palmitic acid C 16:0	9.2	11
Stearic acid C18:0	2.7	4
Oleic acid C18:1 ω-9	27.8	24
Linoleic acid C 18:2 ω-6	18.7	53
α-Linolenic acid C 18:3 ω-3	2.4	8
Stearidonic acid C 18:4 ω-3	0.4	—
Arachidonic acid C 20:4 ω-6	0.5	—
Eicosapentaenoic acid C 20:5 ω-3	2.4	—
Docosapentaenoic acid C 22:5 ω-3	0.3	—
Docosahexaenoic acid C 22:6 ω-3	2.2	—
Others	5.7	—
Ratio ω-6/ω-3	2.5:1	7:1

a Percentage of fatty acids (wt/wt), mean values, reprinted with permission from the manufacturer, Fresenius-Kabi.

Each patient was assigned a patient number at the time of inclusion, and the amount of fat for infusion was calculated. During the prestudy period (day 0), signed parents’ informed consent was obtained, eligibility was assessed, and demographic data, medical history, concomitant diseases, and medications of the neonates were recorded. Clinical assessments (heart rate, temperature, blood pressure, body weight, oxygen therapy) were performed daily from day 0 (prestudy visit) until study termination, either on day 15 or following the last infusion of study treatment (posttreatment). Body height was assessed at birth and posttreatment. At the prestudy visit (baseline) and on days 5, 8, and 15 during the study period, blood samples for assessment of triglycerides, other lipid parameters (total, high-density lipoprotein [HDL], and low-density lipoprotein [LDL] cholesterol), electrolytes, glucose, and liver enzymes were drawn. Baseline samples for the assessment of hematological parameters and bilirubin were drawn on day 3 and for creatinine on day 5. AEs and serious adverse events (SAEs) were defined in accordance with the ICH, and their possible relation to study treatment was documented until 1 day after the end of the last study infusion. The reporting period for SAEs ended 6 days after the end of the last study infusion. All subjects were followed up until discharge. Blood samples for assessing plasma and RBC phospholipid fatty acid pattern and plasma phospholipids were drawn at baseline (pretreatment) and 4 hours after the last study infusion (posttreatment). The primary safety parameter was the change in the concentration of serum triglycerides (mmol/L) from baseline to day 8; secondary safety criteria included hematological and clinical laboratory parameters, AE profile, tolerability, and vital signs. Primary efficacy criteria were the change in body weight from study day 1 to day 8 and height from birth length to last observation. Furthermore, days on mechanical ventilation/oxygen therapy and the change in plasma and RBC fatty acid pattern were to be evaluated.

### Analytical Methods

#### Methods of Sample Preparation and Storage

Blood samples were collected by venous puncture or arterial line. All determinations of routine laboratory assessments, including triglycerides, were assessed using established validated standard techniques. Analyses were performed by the Clinical Laboratories of the University Hospitals Leuven. These laboratories undergo regular external validation (Good Laboratory Practice) and evaluation (BELAC, Belgian Accreditation Body).

Blood samples for fatty acid determination were centrifuged (15 minutes, 3500 rpm), and plasma was subsequently separated from the cellular fraction. Following removal of the buffy coat, both the plasma and the RBC fraction were stored at −70°C until analysis. Plasma and RBC fatty acids were assessed by gas chromatography/mass spectrometry and displayed as relative amounts (% of total fatty acids).^19^

### Statistical Analysis

All statistical analyses were performed for the intention-to-treat (ITT) population. Homogeneity of baseline characteristics was evaluated by means of the Wilcoxon 2-sample test (*t* approximation). Within-group comparison of changes from baseline was carried out by means of the 2-sided Wilcoxon signed rank test and based on subjects with pairwise data only; between-groups comparisons were carried out by means of the Wilcoxon test. Results are presented as mean ± SD. The level of significance was set at *P* < .05.

The aim of the primary analysis was to show noninferiority of the test treatment compared to controls. Test of noninferiority was performed using a confidence limit approach applying the 1-sided Wilcoxon-Mann-Whitney test for noninferiority. As a reasonable benchmark, the lower equivalence margin was defined as a Mann-Whitney estimator (MW) = 0.38. If the lower bound (LB) of the 1-sided 95% confidence interval (CI-LB) is >0.38, then noninferiority is proven in a confirmatory way up to this narrow margin.^20,21^

The primary end point was the safety parameter serum triglycerides, which were evaluated as a change from baseline to day 8. In case the study was discontinued before study day 8, missing values were replaced by means of the “last value carried forward” (LVCF) procedure from day 5. Treatment groups were compared by using differences of means and their confidence bounds calculated by least squares means from analysis of variance (ANOVA) with adjustment for baseline and covariates. The primary efficacy criteria of body weight and body length were evaluated as change (g) from day 1 to day 8 (LVCF) and change from birth length (cm) to last observation, respectively. Between-group differences were evaluated by calculating least squares means (ANOVA) of change from baseline adjusted for baseline and covariates.

The time to end artificial or supportive ventilation over the whole treatment period was evaluated descriptively by Kaplan-Meyer curves, and the differences between treatment groups were evaluated using the Peto log-rank test. The Cox regression model was used for the adjustment for stratum/birth weight. Unless indicated otherwise, fatty acids, biochemical and hematological parameters, and vital signs were evaluated as median changes from baseline/day 3 (for hematological parameters) to day 8.

## Results

### Participant Flow

**Figure 1.**
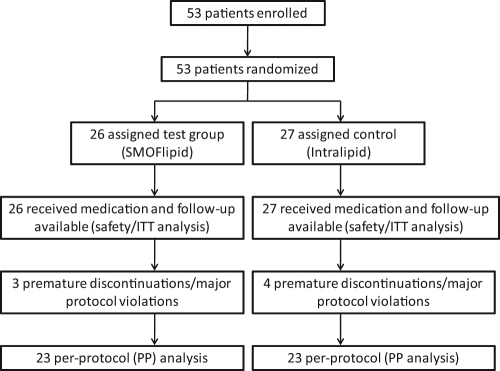
Trial profile and participant flow. The number of patients and the actual study profile are shown in each block. A total of 53 patients were randomized, and all 53 patients were included in the safety and primary efficacy analysis (intention-to-treat population). The per-protocol (PP) analysis comprised 46 patients. ITT, intention to treat.

All 53 neonates received at least 1 dose of study medication and had at least 1 safety assessment afterward. All patients were included in the ITT population, which was identical to the safety population. In total, 16/17 (test/control group) infants had at least 1 protocol deviation. Most frequent reasons included “enteral nutrition with more than 20% of total energy intake at the beginning of lipid supplementation” (12/12), “study treatment compliance not at least 80% in 6 out of 7 treatment days during the main study phase” (3/3), and “premature termination during the main study phase” (2/3); the latter two were regarded as *major* protocol deviations leading to exclusion from the per-protocol (PP) population analysis, which was the case in 7 patients (3/4). Reasons for exclusion were “not compliant” (1/1) “adverse event and not compliant” (1/1), “worsening of disease and not compliant” (1/0), “worsening of disease and adverse event” (0/1), and “consent withdrawn and not compliant” (0/1). An overview of the participant flow is presented in Figure 1.

Importantly, infants’ recovery during the main study (no need for further PN) was not regarded as a major protocol violation. The PP data set comprised 46 infants. According to the definition of the study period (minimum 7 days, up to 14 days), the number of study patients decreased rapidly after study day 7. A main study phase was defined as the period from the day of inclusion until completion of treatment day 7 (baseline to day 8) and the complete study phase up to day 15 as a maximum.

### Baseline Data

Baseline characteristics were comparable between the treatment groups (Table 4); the only significant difference was a markedly higher rate of female neonates in the test group as compared to the control group (69.2% vs 40.7%; *P* = .054). Infants in the test group were slightly more immature with regard to gestational age, body weight, and length and the age at the start of infusions. Both treatment groups were comparable with respect to physical examination results at baseline and previous medication (data not shown). Main diagnoses in the study/control group were “respiratory distress syndrome” in 21/22 infants and “difficulties to adaptation,” meaning transient tachypnea of the newborn, in 18/17 infants. Treatment groups did not differ with respect to the exposure to study drug and nutrient intake during the main and complete study phase (Table 5), and total parenteral fat intake per kg body weight was in accordance with the study protocol. Maximal parenteral fat intake was 3.4 g/kg BW/d in the SMOFlipid group and 3.3 g/kg BW/d in the Intralipid group. Two infants received PN exclusively. For the other patients, the enteral nutrition (EN) part of the nutrition consisted of expressed breast milk in 15 patients, formula in 13 patients, and combined expressed breast milk and formula in 23 patients.

**Table 4.** Overview of Baseline Demographic and Clinical Characteristics: Intention-to-Treat Population

	n	Test Group (SMOFlipid)	n	Control Group	*P* Value (Test of Homogeneity)^a^
Gestational age, wk, mean (SD)	26	29.9 (1.9)	27	30.4 (1.8)	.195
Age at start of infusion, d, mean (SD)	26	7.0 (1.4)	27	7.3 (2.0)	.464
Gender, % of females	26	69.2	27	40.7	.054^b^
Birth length, cm, mean (SD)	25	38.9 (3.8)	25	39.1 (3.2)	.371
Birth weight, g, mean (SD)	27	1335.6 (408.8)	27	1364.1 (339.7)	.440
Head circumference, cm, mean (SD)	25	27.6 (2.7)	23	27.5 (2.0)	.356
Apgar score, 60 seconds, No. (%)					.683^c^
>8	26	11 (42.3)	27	9 (33.3)	
6–8	26	10 (38.5)	27	13 (48.1)	
<6	26	5 (19.2)	27	5 (18.5)	
Apgar score, 300 seconds, No. (%)					.885^c^
>8	26	16 (61.5)	27	15 (55.6)	
6–8	26	9 (34.6)	27	12 (44.4)	
<6	26	1 (3.8)	27	0	

a Wilcoxon 2-sample test (*t* approximation).

b Fisher exact test.

c Mantel-Haenszel test.

**Table 5.** Exposure to Study Treatment and Nutrient Intake: Intention-to-Treat Population, With No Significant Group Differences

	At Study Start (Day 1)				Main Study Phase^a^				Complete Study Phase^b^			
	n	Test Group (SMOFlipid)	n	Control Group	n	Test Group (SMOFlipid)	n	Control Group	n	Test Group (SMOFlipid)	n	Control Group
Daily parenteral glucose intake, g/kg BW/d	26	14.3 (3.0)	27	15.3 (1.5)	26	12.5 (3.1)	27	13.2 (1.7)	26	12.2 (2.8)	27	12.6 (1.5)
Daily parenteral amino acid intake, g/kg BW/d	26	2.5 (0.5)	27	2.7 (0.3)	26	2.2 (0.5)	27	2.3 (0.3)	26	2.2 (0.5)	27	2.2 (0.3)
Daily parenteral fat intake, g/kg BW/d	26	1.0 (0.2)	27	1.0 (0.2)	26	2.0 (0.4)	27	2.0 (0.4)	26	2.1 (0.5)	27	2.1 (0.5)
Daily enteral fat intake, g/kg BW/d	23	0.42 (0.37)	24	0.31 (0.20)	24	0.76 (0.41)	26	0.70 (0.41)	24	0.82 (0.36)	27	0.77 (0.36)
Daily parenteral + enteral fat intake, g/kg BW/d	26	1.42 (0.47)	27	1.32 (0.24)	26	2.69 (0.48)	27	2.61 (0.68)	26	2.87 (0.42)	27	2.87 (0.62)
Daily enteral/total fat intake ratio	23	0.25 (0.15)	24	0.21 (0.11)	24	0.27 (0.15)	26	0.24 (0.11)	24	0.28 (0.14)	27	0.25 (0.10)

Values presented as mean (SD). BW, body weight.

a Defined as the period from the day of inclusion until completion of treatment day 7.

b Defined as the period from the day of inclusion until termination of study treatment application.

### Primary Outcome Measures

On day 8, triglyceride levels were increased in both treatment groups vs baseline (see Table 6) and were significant in the test group only (*P* < .05). Yet, there was no significant difference between treatment groups with regard to the mean increase in triglycerides from baseline to day 8/LVCF (0.18 ± 0.35 vs 0.09 ± 0.33 mmol/L corresponding to 15.93 ± 30.97 vs 7.96 ± 29.20 mg/dL, test vs control, *P* = .702, Wilcoxon test). Accordingly, least squares means of change from baseline showed no noteworthy differences between groups (ITT) when adjusted for baseline and covariates (age, stratum, Apgar score). The test result for the confirmatory analysis was MW = 0.4659 [0.3119; 0.6267]. Because the lower limit of the confidence interval was below the lower equivalence margin of 0.38, according to the benchmarks defined by Colditz et al,^22^ noninferiority of the study treatment could not be demonstrated in a confirmatory sense.

**Table 6.** Clinical Laboratory and Lipid Parameters at Baseline and on Day 8 (After the Main Study Phase): Intention-to-Treat Population

	Baseline				Day 8				*P*^a^	*P*^b^	
	n	Test Group (SMOFlipid)	n	Control Group	n	Test Group (SMOFlipid)	n	Control Group	Test vs Control	Test Group (SMOFlipid)	Control Group
Hemoglobin, g/dL^c^	25	13.0 (1.87)	24	12.9 (2.08)	12	11.8 (2.02)	13	12.1 (2.16)	.276	.001	.002
Hematocrit, %^c^	25	36.6 (4.79)	24	36.4 (5.22)	12	33.5 (5.56)	13	34.9 (5.94)	.468	.003	.002
Red blood cells, 10^12^/L^c^	25	3.7 (0.51)	24	3.6 (0.52)	12	3.4 (0.53)	13	3.5 (0.50)	.235	.003	.002
White blood cells, 10^9^/L^c^	25	14.6 (5.16)	24	12.0 (3.18)	12	12.7 (2.43)	13	9.8 (3.14)	.176	.029	.322
Platelets, 10^9^/L^c^	25	406.2 (133.27)	24	370.5 (144.75)	12	477.1 (116.49)	13	454.5 (176.65)	.817	.007	.084
ALT, IU/L	25	6.0 (1.57)	25	6.4 (1.64)	21	9.8 (4.83)	21	9.2 (4.11)	.888	.003	.008
GGT, IU/L	24	89.8 (55.37)	19	71.0 (47.42)	21	73.1 (33.99)	18	63.0 (44.30)	.135	.126	.465
Direct bilirubin	19		22		19		22		.036	.347	.022
µmol/L^c, d^		12.48 (7.2)		8.04 (5.6)		10.26 (5.8)		12.83 (7.2)			
mg/dL		0.73 (0.42)		0.47 (0.33)		0.6 (0.34)		0.75 (0.42)			
Total bilirubin	26		27		19		24		.049	<.001	.137
µmol/L^c, d^		127.6 (70.82)		115 (47.26)		94.8 (68.18)		98.2 (74.69)			
mg/dL		7.46 (4.14)		6.73 (2.76)		5.54 (3.99)		5.74 (4.37)			
Creatinine^e^	25	70.9 (12.36)	26	70.7 (10.45)	16	63.3 (4.83)	13	67.3 (8.09)	.614	.003	.003
CRP, mg/L	26	1.4 (1.09)	27	2.0 (3.42)	21	2.3 (5.89)	22	9.2 (37.90)	.965	.438	.234
Glucose, mmol/L	25	5.4 (2.80)	25	4.8 (0.94)	23	5.0 (0.88)	22	4.9 (0.88)	.827	.571	.480
Serum triglycerides	26		23		20		22		.781	.027	.072
mmol/L		0.52 (0.16)		0.54 (0.19)		0.69 (0.38)		0.67 (0.36)			
mg/dL		46.02 (14.16)		47.79 (16.81)		61.06 (35.40)		59.29 (30.10)			
Total cholesterol, mmol/L	26	3.1 (0.91)	22	3.1 (0.90)	12	3.9 (0.86)	13	3.0 (0.74)^f^	.060	.034	.652
Ratio LDL/HDL cholesterol, g/cm (IU/L)	24	1.22 (0.59)	22	1.18 (0.54)	12	1.89 (0.68)	13	1.48 (0.64)	.160	.014	.820
HDL cholesterol, mmol/L	25	1.4 (0.51)	23	1.4 (0.44)	12	1.3 (0.25)	13	1.1 (0.28)	.742	.182	.232
LDL cholesterol, mmol/L	24	1.5 (0.70)	22	1.5 (0.61)	12	2.3 (0.73)	13	1.6 (0.64)	.080	.040	1.000

Values presented as mean (SD). All comparisons of change from baseline (within and between groups) are based on patients with pairwise available data. ALT, alanine 3aminotransferase; CRP, C-reactive protein; GGT, glutamyl transpeptidase; HDL, high-density lipoprotein; LDL, low-density lipoprotein.

a Between-groups comparison of change from baseline, Wilcoxon test.

b Within-groups comparison (baseline vs day 8), Wilcoxon signed rank test.

c Baseline value = day 3.

e Baseline value = day 5.

d Final value = last visit.

f Between-groups comparison on day 8, *P* < .05.

On day 8, body weight was significantly increased vs baseline in both treatment groups (baseline/day 1: 1359 ± 311 g vs 1278 ± 295 g; day 8: 1394* ± 355 g vs 1381* ± 330 g; test vs control; *P < .01 day 8 vs baseline within group). Changes from baseline were similar between groups (95.18 ± 123.81 vs 118.54 ± 84.33 g, test vs control, *P* = .59). Daily weight gain, depending on the respective body weight up to day 8, was not different between groups (13.0 ± 25.5 vs 15.7 ± 11.1 g/kg BW, test vs control, *P* = .30).

Body height increased slightly but significantly in both treatment groups over the complete study phase (baseline: 38.9 ± 3.8 cm vs 39.1 ± 3.2 cm; last observation: 40.7* ± 3.8 cm vs 40.7* ± 3.3 cm; test vs control; *P < .01 last observation vs baseline within group). Changes from baseline were similar between groups (*P* = .89). Least squares means of change from baseline showed no noteworthy differences between groups with regard to body weight as well as to body length (ITT) when adjusted for baseline and covariates (age, stratum). Analysis of the PP population (data not shown) revealed the same pattern as for the ITT population.

### Secondary Outcome Measures

Mean values for hemoglobin, hematocrit, and red and white blood cell count showed a decrease and platelets an increase in both groups after the main study phase (Table 6). Yet, values for changes from baseline to day 8 were not significantly different between treatment groups, and mean values remained within the normal range with no clinical relevancy. Evaluation of lipid parameters revealed no significant differences between groups at baseline and on study day 8, with the exception of total cholesterol and LDL cholesterol on study day 8, which were higher in the test group (*P* < .05 between-groups comparison of change from baseline, Wilcoxon test) but still remained within normal ranges (Table 6). Clinical laboratory parameters changed significantly over the main study phase within both groups for alanine aminotransferase (ALT; significant increase in both treatment groups) and creatinine (significant decrease in both treatment groups). In the test group, a significant decrease in total and a slight decrease in direct bilirubin levels were observed (Table 6). In controls, direct bilirubin levels increased significantly from baseline to the final observation. For total and direct bilirubin levels, the absolute change from baseline to last observation was significantly different between groups (total bilirubin: −50.3 ± 45.8 vs −18.6 ± 54.2 µmol/L corresponding to −2.94 ± 2.68 vs −1.09 ± 3.17 mg/dL; direct bilirubin: −2.22 ± 8.89 vs 4.79 ± 8.38 µmol/L corresponding to −0.13 ± 0.52 vs 0.28 ± 0.49 mg/dL, test vs control, *P* < .05 between-groups comparison of change from baseline, Wilcoxon test).

In total, 75 AEs in 29 patients (11 study group/18 control group) were observed during the treatment period—32 in the study and 43 in the control group—affecting most frequently the system organ class “infections and infestations” (3/8), including chorioamnionitis, bacteremia with or without sepsis, respiratory tract infections, and conjunctivitis; “hepatobiliary disorder” (4/6), including increased total and indirect serum bilirubin necessitating treatment (fototherapy or albumin replacement) and increase of direct serum bilirubin (>17.1 µmol/l and >20% of total bilirubin), serum glutamyl transpeptidase (GGT), and ALT that exceeded normal biochemical limits; and “metabolism and nutrition disorders” (4/6), including hyperglycemia and metabolic acidosis. Most AEs were of mild and moderate intensity; 6 SAEs were experienced in the study group and 5 in the control group.

During the treatment period, 4 infants (2 in each treatment group) experienced serious treatment-emergent AEs (teSAEs) leading to discontinuation of study medication, with 2 of them resulting in the death of the patient (pneumothorax, test group; enterobacter sepsis, control group). Further teSAEs observed included hyperglycemia (test group) and staphylococcal sepsis (control group). Another 2 patients (1 in each treatment group) experienced posttreatment SAEs, one of them (multiorgan failure, control group) resulting in the death of the patient. Almost all SAEs in both treatment groups were assessed as “not related to study drug,” with 1 exception of enterobacter sepsis in the control group, which was rated as “possibly” related to the study drug. Apart from the 2 infants who died during treatment and 1 who died in the posttreatment phase, all others survived until discharge.

The tolerability of the study treatment was assessed as being “very good” or “good”; there was no difference between treatment groups. An effect of study medication on vital signs (blood pressure, heart rate, body temperature) pre- and posttreatment was not observed (data not shown).

In the test group (n = 26), the number of infants receiving supportive or artificial ventilation was 16 during the main study phase and 18 over the complete treatment period. In controls (n = 27), 18 infants received supportive/artificial ventilation during both study phases. Median time to end of supportive or artificial ventilation was 2 vs 3 days (test group vs control group; ITT). Yet, superiority of test treatment could not be shown in the Peto log-rank test (*P* = .7986) or the Cox regression model (*P* = .8239). Analysis of the PP population (data not shown) showed no significant differences to the ITT population.

**Figure 2.**
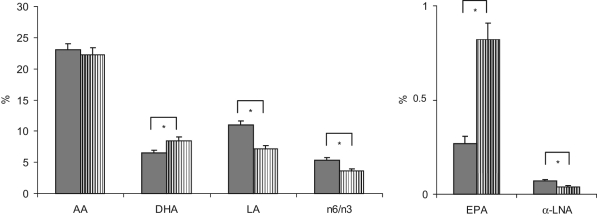
Fatty acid pattern in red blood cells after study treatment (% of total fatty acid, mean ± SEM). Striped bars: SMOFlipid, solid bars: control. *P < .05 between groups (Wilcoxon test). AA, arachidonic acid; α-LNA, α-linolenic acid; DHA, docosahexaenoic acid; EPA, eicosapentaenoic acid; LA, linoleic acid.

Mean baseline values of plasma lipoprotein phospholipid fatty acids shown as relative amounts were very similar in both treatment groups (*P* > .05, Wilcoxon test). Fatty acid patterns in plasma phospholipids pre- and posttreatment and in RBC phospholipids posttreatment are shown in Table 7 and Figure 2, respectively.

**Table 7.** Fatty Acid Pattern (% of Total Fatty Acids) in Plasma Phospholipids Before and After Study Treatment: Intention-to-Treat Population

	Pretreatment				Posttreatment				*P*^a^	*P*^b^	
	n	Test Group (SMOFlipid)	n	Control Group	n	Test Group (SMOFlipid)	n	Control Group	Test vs Control	Test Group (SMOFlipid)	Control Group
C18:1 n-9 (oleic acid)	22	29.84 (4.66)	23	28.04 (4.31)	19	25.72 (2.87)	20	19.48 (2.92)^c^	.043	.009	<.001
C20:3 n-9	22	1.32 (0.76)	23	1.35 (1.11)	19	0.16 (0.30)	20	0.05 (0.06)^c^	.987	<.001	<.001
Total n-6 PUFA	22	12.45 (6.49)	23	12.34 (5.51)	19	35.36 (4.54)	20	48.08 (5.81)^c^	<.001	<.001	<.001
C18:2 n-6 (LA)	22	3.88 (2.82)	23	3.73 (2.33)	19	29.02 (4.92)	20	41.78 (6.66)^c^	<.001	<.001	<.001
C20:4 n-6 (AA)	22	6.99 (4.17)	23	7.02 (3.46)	19	5.07 (1.17)	20	5.30 (1.55)	.477	.263	.036
Total n-3 PUFA	22	1.96 (0.90)	23	1.65 (0.71)	19	4.44 (1.29)	20	2.24 (0.41)^c^	<.001	<.001	.008
C18:3 n-3 (α-LNA)	22	0.03 (0.03)	23	0.02 (0.02)	19	0.24 (0.09)	20	0.44 (0.20)^c^	<.001	<.001	<.001
C20:5 n-3 (EPA)	22	0.14 (0.08)	23	0.13 (0.07)	19	1.35 (0.64)	20	0.13 (0.07)^c^	<.001	<.001	.898
C22:6 n-3 (DHA)	22	1.72 (0.79)	23	1.44 (0.62)	19	2.50 (0.70)	20	1.50 (0.37)^c^	.002	.001	.609
n-6/n-3 ratio	22	6.67 (2.41)	23	7.65 (2.10)	19	8.50 (2.29)	20	22.04 (4.37)^c^	<.001	<.001	<.001

Values presented as mean (SD). All comparisons of change from baseline (within and between groups) are based on patients with pairwise available data. AA, arachidonic acid; α-LNA, α-linolenic acid; DHA, docosahexaenoic acid; EPA, eicosapentaenoic acid; LA, linoleic acid; PUFA, polyunsaturated fatty acid.

a Between-groups comparison of change from baseline, Wilcoxon test.

b Within-groups comparison (pre- vs posttreatment), Wilcoxon signed rank test.

c Between-groups comparison on day 8, *P* < .05.

## Discussion

Premature neonates have only limited muscle and fat mass and thus decreased hydrolytic capacity of the enzyme lipoprotein lipase.^23^ As a consequence, they are at higher risk for PN-associated hypertriglyceridemia than term infants.^1,2^ Tight monitoring of serum triglyceride levels has been recommended in premature neonates to avoid hypertriglyceridemia with the provision of increasing lipid concentrations as recommended by actual PN guidelines.^2^ Therefore, our primary hypothesis was that, in premature neonates, test treatment would not be inferior to a standard soybean oil emulsion with regard to the serum concentration of triglycerides.

Throughout the study, mean serum triglyceride levels remained below the upper limit of normal range (1.6 mmol/L or 142 mg/dL) in both treatment groups for all time points (baseline, day 5, day 8, post day 9, and last visit). For the present trial, the benchmark of clinical relevance was set at 3.43 mmol/L (304 mg/dL). In the meantime, recommendations of ESPEN/ESPGHAN have become available that consider a concentration of serum triglycerides exceeding 2.82 mmol/L (250 mg/dL) a critical limit calling for a reduction in parenteral lipid dose.^2^

As a result of the lipid infusion, there was a slight increase in triglycerides from baseline to day 8 in both treatment groups, which was significant in the test group (*P* = .03) and tended to be significant in the control group (*P* = .07) but was not different between groups (*P* = .15). Yet, statistical proof of the noninferiority of the study treatment could not be given in a confirmatory sense. Possibly, the fact that infants in the test group were slightly more immature than control infants might have influenced the tolerance to parenteral lipids, explaining the failure to give confirmatory proof of the equality of treatments administered. Yet, with regard to the critical limits, the slight and comparable increase in triglyceride concentrations as observed with both study treatments (<0.2 mmol/L) may be rated of no clinical relevance, confirming the safety of both treatments administered. Triglyceride concentrations observed in the present trial are also in line with those reported by Tomsits et al^24^ in a similar study population, supporting their conclusion that a physical mixture of soybean oil, MCTs, olive oil, and fish oil is safe and well tolerated in premature infants requiring PN.

The significant increase in LDL cholesterol in the test group as compared to the controls is not well explained and is in contrast with the results obtained in the study by Tomsits et al.^24^ In this study, no differences in HDL and LDL cholesterol were observed. In the present study, the values of the LDL/HDL ratio are comparable with breastfed preterm infants.^25^

For the hematological parameters, changes observed after study treatment in both groups might be ascribed both to the routine blood sampling and to the physiological anemia frequently experienced by preterm babies.^26,27^ Clinical laboratory parameters showed no clinically relevant changes during the main study phase. Predominantly, values reflect the common postnatal development in this patient population (eg, maturation of kidney function). Consequently, other safety aspects, such as hematological and clinical laboratory parameters, as well as the adverse event profile and evaluation of vital signs indicate that both lipid emulsions were equally safe and well tolerated over 7–14 days.

Premature neonates with low birth weights, receiving PN for longer periods, are at an enhanced risk to develop cholestasis and PN-associated liver disease (PNALD).^1,28-31^ Monitoring of total and, in particular, direct bilirubin levels during parenteral feeding is thus crucial. In the present trial, mean values for direct bilirubin remained below the critical level of 17.1 µmol/L and <20% of total bilirubin, respectively, before and after study treatment.^31^ Total bilirubin levels decreased significantly in the neonates receiving the test mixed emulsion after 7–14 days of parenteral feeding but not in controls receiving a soybean oil–based emulsion. Furthermore, although a slight decrease in direct bilirubin could be seen in the test group over the treatment period, this parameter showed a significant increase in controls. Short-term application of the new lipid emulsion was thus associated with beneficial effects on serum bilirubin levels in premature neonates, thereby potentially reducing their risk of developing cholestasis.

The concept that a change in parenteral lipid regimen, from a predominance of n-6 fatty acids to lipid emulsions containing n-3 fatty acids from fish oil, may be effective in the prevention and treatment of PNALD is still under discussion.^17,32-34^ Beneficial effects on liver function with a mixed emulsion containing soybean oil, MCT, olive oil, and fish oil were observed in a few studies in adult and pediatric patients: in adult intensive care unit patients after major surgery, a lower rise in liver enzymes and in the phospholipids/plasma apolipoprotein A1 ratio (a surrogate marker of liver function) suggested better liver function by PN with the test emulsion than with a soybean oil emulsion.^35^ In postoperative surgical patients, ALT, aspartate aminotransferase, and α-glutathione S-transferase levels (markers of hepatic integrity) were also significantly lower with the test emulsion as compared to a lipid emulsion based on olive and soybean oils, indicating a better liver tolerability.^36^ In a study in children receiving long-term PN with the test emulsion, a significant improvement in plasma bilirubin could be observed.^37^

The present findings must be interpreted with caution, though, regarding possible benefits of the test lipid emulsion on the development of PNALD in premature infants. First, the observed reduction in total bilirubin, to some extent, reflects the common postnatal development in this patient population. Furthermore, patients need to be on PN for a longer time before negative effects on liver function become apparent, whereas most of the neonates in the present trial did not require PN beyond the third week of life. Further trials evaluating the potential hepatoprotective effects of the new lipid emulsion over prolonged periods on a larger number of patients are needed.

Soybean oil–based lipid emulsions contain high amounts of γ-tocopherol but relatively low amounts of α-tocopherol, the main lipophilic antioxidant. In some older studies, infusion of soybean- or safflower–based lipid emulsions has been associated with an increased production of peroxidative intermediates and, therefore, an aggravated risk of oxidative stress.^38,39^ Oxidative stress, representing a common mediator of the inflammatory process, has been associated with hepatocellular injury in preterm infants on PN.^40^ In previous studies evaluating the test emulsion in preterm infants and children, parameters of lipid peroxidation did not differ between treatment groups, but vitamin E status and total antioxidant potential were significantly improved with the provision of adequate amounts of vitamin E as compared to controls receiving a soybean oil emulsion.^24,37,41^ Unfortunately, measurements of vitamin E status and lipid peroxidation were not within the scope of the present investigation. However, with regard to the previous finding, it was assumed also in the present trial that the provision of increased amounts of α-tocopherol with the test emulsion may have contributed to protecting the liver against PN-induced peroxidative damage. Eventually, the partial replacement of soybean oil with fish oil in the new lipid emulsion resulted in a lower phytosterol intake in the infants receiving the test emulsion. It has repeatedly been suggested that phytosterols may represent a further contributing factor to the pathogenesis of PN-related cholestasis.^30,42-44^ Phytosterol concentrations have been shown to be particularly high in plant oil–based lipid emulsions, whereas fish oil emulsions are free from phytosterols.^35^

In our study, fatty acid profiles were to be measured in plasma and in RBC phospholipids pre- and posttreatment. Unfortunately, reliable baseline values for RBC fatty acid composition are not available because of difficulties in sample handling that occurred during the isolation of RBCs from the blood samples drawn at baseline. Therefore, only posttreatment results are presented for RBC phospholipid fatty acid composition.

In both plasma and RBCs, values measured for the essential fatty acids LA and α-LNA posttreatment were significantly higher in the group receiving the soybean oil emulsion than in the infants receiving the test emulsion. These findings reflect the different fatty acid composition of the investigational lipid emulsions, thereby confirming compliance to the treatments administered.

Also, the long-chain homologues of α-LNA (ie, EPA and DHA) were significantly increased with the mixed fish oil containing test emulsion as compared to controls’ posttreatment values. Obviously, the increased provision of EPA and DHA with the fish oil component of the tested emulsion more than outweighed the decreased supply of the precursor fatty acid in both compartments. The increased values for EPA and DHA observed in the present study are in line with the findings by Goulet et al^37^ in pediatric patients on long-term PN showing significantly increased relative amounts of both EPA and DHA in plasma and RBC phospholipids after 28 days. Although Tomsits et al^24^ also recently reported an increase in RBC-EPA in premature neonates receiving the test emulsion, they did not observe a corresponding increase in RBC-DHA. The reason for this finding could be that the preterm neonates in that study received up to a maximum of 2 g lipids/kg BW/d. In a recent study of preterm infants <1250 g receiving a lipid emulsion containing 10% fish oil, a decrease of DHA in RBC was noticed.^45^ However, this was less significantly decreased compared to the group receiving a soybean oil–based emulsion.

The significance of these findings has to be related to the intrauterine and postnatal accretion of LC-PUFAs in preterm and term infants. During the third trimester of fetal life, a rapid accretion of DHA, EPA, and AA takes place.^46,47^ Because of the relatively inefficient endogenous conversion of α-LNA to DHA in the newborn, there seems to be an apparent need for the supplementation of preformed LC-PUFAs in preterm infants.^5^ It has been calculated that the need for supplementation of DHA in preterm infants is even higher than actually provided by the supplemented preterm formulas.^48^ It has been suggested that enteral feeding supplemented with DHA is beneficial for the neurodevelopmental outcome of preterm infants.^49^ Furthermore, it could be hypothesized that measurements of RBC phospholipids allow predictions of this accretion and its potential beneficial effect on visual acuity and mental and psychomotor development in later infancy.^50^ Indeed, an adequate intake of dietary DHA and AA seems to be required for optimal functional maturation of the retina and visual cortex.^11,51-53^ It is also generally accepted that LC-PUFAs, particularly the n-3 LC-PUFA from fish oil, possess potent immunomodulatory and anti-inflammatory properties.^54^ A shift from a predominance of n-6 fatty acids to n-3 LC-PUFAs from fish oil in PN may be effective in modulating the eicosanoid profile toward a reduction in circulating levels of proinflammatory mediators, thereby potentially contributing to decreasing the risk of hepatic injury.^30^ In the present trial, C-reactive protein levels showed an increase over the main study period in both groups, which was distinctly yet not significantly more pronounced in the infants receiving the soybean oil emulsion.

In plasma and RBC phospholipids, posttreatment values for the long-chain homologue of LA, arachidonic acid (AA, C20:4 n-6), were comparable with both lipid emulsions, indicating that small amounts of AA (0.5%) and LA (18.7%) contained in the test emulsion efficiently compensated for the much higher amount of LA (53%) contained in the soybean oil emulsion. AA has been shown to affect growth and development in early infancy in a positive way.^11^ Furthermore, the supply of AA in preterm infants has been associated with the weight at birth and growth during the first year of life.^55^ In the present trial, both body weight and height were significantly increased vs baseline in both treatment groups, independent of lipid source, suggesting that PN with SMOFlipid, providing n-3 fatty acids from fish oil, was equally effective in promoting growth in premature neonates as a conventional soybean lipid emulsion. This finding might be of particular interest, taking into account that 2 trials published in the 1990s have raised concerns that the use of a fish oil–enriched enteral formula might reduce the growth of preterm infants.^56,57^ It has been shown, however, that the decrease in weight gain was due to a deficiency in AA in the fish oil–supplemented formula and that optimal weight gain requires a well-balanced DHA/AA. This was confirmed in a recent Cochrane Database Systematic Review showing that formula supplemented with DHA and AA, with or without EPA, did not impair growth.^58^ Consequently, recommendations have been made about the supply of LC-PUFAs with infant formula.^59,60^ Fish oil–based emulsions for PN naturally contain both EPA and DHA. The ESPGHAN guidelines on neonatal and pediatric PN have no recommendations on the individual LC-PUFAs.^2^ The increase of EPA observed in this study may be of concern. In a long-term feeding study with a formula high in linolenic acid and marine oil (without additional AA), plasma and RBC values of EPA were far above the values found in the present study.^61^ The follow-up of those children has been the subject of a number of studies, with the general conclusion that apart from an impaired growth (AA deficiency), the group with high marine oil supplementation had a improved visual and psychomotor development at 12 months compared to the group with standard formulation and comparable with breastfed infants.^62-64^ A similar increase of EPA in plasma and red blood cells has been found in a recent study in preterm neonates <1250 g receiving a lipid emulsion containing 10% fish oil.^45^

The n-6/n-3 fatty acid ratio was significantly lower with the test emulsion as compared to controls posttreatment. This reflects the n-6/n-3 fatty acid ratio in the test emulsion corresponding to approximately 2.5:1, which is in accordance with acknowledged recommendations for adults and approximates the ratio in human milk.^49,65,66^ In contrast, for soybean lipid emulsions, an n-6/n-3 fatty acid ratio of 7:1 has been reported.^65^ In line with the present data, the authors of a clinical study in surgical patients have reported significantly higher total n-3 and lower total n-6 fatty acids in plasma phospholipids after 5 days of PN, including the mixed fish oil containing test emulsion, resulting in a decreased n-6/n-3 fatty acid ratio as compared to a standard soybean oil emulsion.^67^ A modification of the RBC phospholipid pattern, as indicated by a significantly lower n-6/n-3 fatty acid ratio, could be seen in another randomized controlled trial in preterm neonates receiving the test emulsion vs a conventional lipid emulsion and in children on long-term PN.^24^

As for many clinical studies in a sensitive population of preterm infants, this study has a number of shortcomings in addition to the reduced number of patients included. A potential source of bias for the present trial rests in the fact that neonates in both treatment groups received roughly 30% of their total fat intake from enteral supplementation. Yet, the results of the present study show that changes in the fatty acid profiles occurred considerably faster with PN as compared to the findings of investigations with oral supplementation.^50^ Possibly, the parenteral route might be predominant in comparison to the enteral route in achieving relevant increases in LC-PUFAs within such a short period. Further investigations are requested to investigate how long these effects can be maintained after the switch from PN to EN in this patient setting. A further potential source of imprecision might be ascribed to the fact that the number of study patients decreased rapidly after treatment day 7. Therefore, study variables were evaluated for the main study phase but also until completion of PN, with the means of the last observation carried forward (LOCF), which allowed evaluating the effect of the different lipid emulsions in most patients. Furthermore, evaluation of baseline characteristics revealed that the rate of females in the test group was higher as compared to the control group (69.2% vs 40.7%; *P* = .054). According to clinical experience, preterm female neonates generally have a more favorable clinical outcome in neonatal care as compared to preterm males. On the other hand, neonates in the test group were slightly more immature with regard to gestational age, body weight, length, and the age at the start of infusions. Consequently, these factors of baseline inhomogeneity might have outweighed each other with regard to potential bias. Finally, the short study period does not allow drawing conclusions on safety when PN is used for longer periods in preterm infants.

In conclusion, the new lipid emulsion, based on a physical mixture of soybean oil, MCT, olive oil, and fish oil, was well tolerated and safe in terms of clinical laboratory and hematological parameters, AE profile, and vital signs over an infusion period of 7–14 days in preterm infants. This emulsion seems to be equally effective in providing a balanced fatty acid and effective energy supply. The primary variable, triglyceride levels, was considered safety related, and the study revealed no statistical difference between the groups, although noninferiority on the basis of serum triglycerides could not be shown in a confirmatory way. Administration of the test lipid emulsion was associated with significantly reduced total and slightly reduced direct bilirubin levels, indicating a potential beneficial effect of SMOFlipid in terms of cholestasis. Furthermore, infusion of the test lipid emulsion was equally efficient as a standard soybean emulsion in promoting the neonates’ growth while being associated with a modification of the plasma and RBC fatty acid pattern reflecting the fatty acid composition of the novel lipid emulsion. The higher availability of n-3 LC-PUFAs with this emulsion might be considered advantageous with regard to their postulated role in ensuring adequate brain and retinal development in preterm infants and the postulated anti-inflammatory and immunomodulatory role. Further studies on longer use and on a larger population are needed in preterm infants to confirm the safety and efficacy, as well as its effect on mental and visual development of this novel lipid emulsion in neonatal care.
